# Supplementary material for: Affiliative zygomatic synchrony in co-present strangers
Source: Sci Rep. 2019 Feb 28;9:3120. doi: 10.1038/s41598-019-40060-4 (PMC6395718; doi:10.1038/s41598-019-40060-4)
Supplement: Supplementary file 1 — Supplementary Figures [file 41598_2019_40060_MOESM1_ESM.pdf]

Affiliative zygomatic synchrony in co-present strangers

Golland Yulia[1,*], Dana Mevorach[1], Nava Levit-Binnun[1]

1. Sagol Center for Brain and Mind, Baruch Ivcher School of Psychology, Interdisciplinary Center, Herzliya, Israel

Supplementary Material

A. Zygomatic synchrony in random dyads (control sampling distributions) and in real dyads (experimental means)

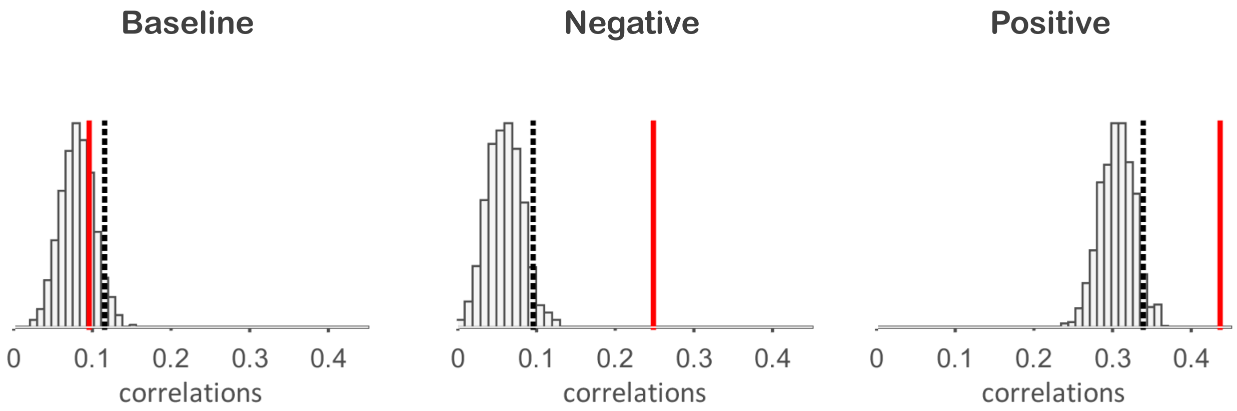

B. Cardiovascular synchrony in random dyads (control sampling distributions) and in real dyads (experimental means)

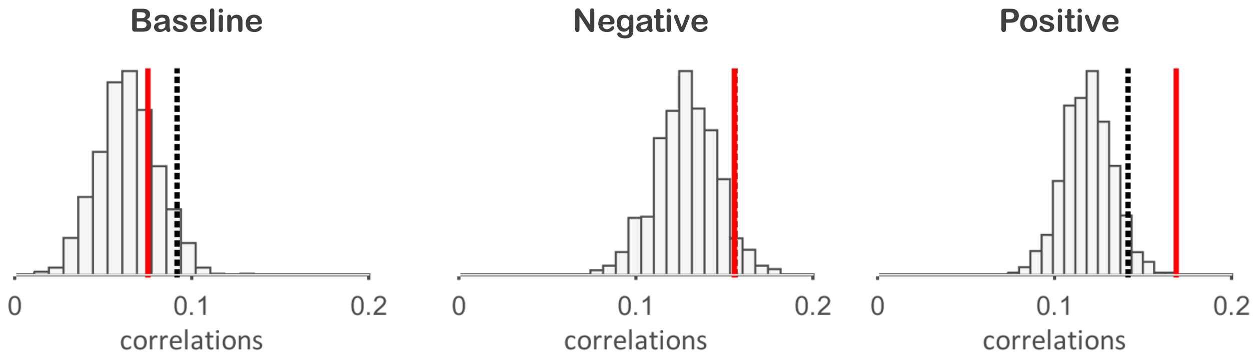

Supplementary Figure 1. A. Zygomatic synchrony in random and in real dyads. Figure presents the results of non-parametric bootstrapping procedure comparing zygomatic synchrony in real and in random dyads. Control sampling distributions of random dyads' synchrony were empirically constructed for each experimental condition, using bootstrapping procedure with 1000 iterations. Dashed lines represent significance threshold (marking 95% of control distributions). Red lines represent sample means of real dyads synchrony. As can be seen from the figure, zygomatic synchrony in real dyads was significantly higher than the one in random dyads during both emotional movies, but not during non-emotional baseline. **B. Cardiovascular synchrony in random and in real dyads.** Similar approach was applied for the indexes of cardiovascular synchrony in real and random dyads. Co-viewing participants showed higher synchrony in their heart rate responses as compared to random dyads participants, during both emotional movies, but not during baseline.

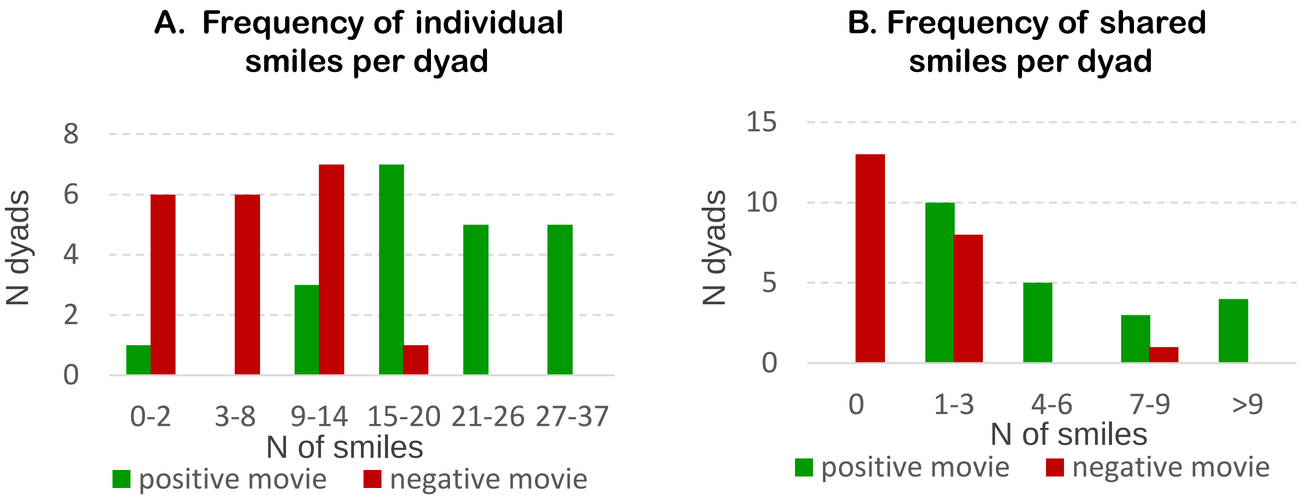

Supplementary Figure 2. A. Frequency of individual smiles per dyad. Frequency of individual smiles (that is smiles exhibited by only one of the co-viewing participants) in a dyad was coded from video recordings of participants' faces. Figure presents frequency of such smiles during the positive and the negative movies. As evident from the figure, while participants smiled to a much higher degree during the positive movie, a substantial number of dyads exhibited smiling behavior during the negative movie as well. **B. Frequency of shared smiles per dyad.** Shared smiles were identified as smiles in both co-viewing participants, initiated within less than 3 sec lag. As evident from the figure, some of the smiling was contagious both during the positive and during the negative movies.
